# Updates in Hypertension Studies According to the Main Clinical Trials: A Review of the Past 45 Years about Pharmaceutical Intervention Effects

**DOI:** 10.3390/nursrep10010002

**Published:** 2020-09-08

**Authors:** Jose Manuel Martínez-Linares

**Affiliations:** Department of Nursing, Universidad de Jaén, 23071 Jaén, Spain; jmlinare@ujaen.es; Tel.: +34-953213676

**Keywords:** arterial hypertension, clinical trial, review, pharmacological treatment, nursing

## Abstract

Background: High blood pressure remains one of the most important risk factors for cardiovascular disease. Although there is no consensus, all the clinical practice guidelines agree on the need to reduce blood pressure levels to minimize the risks. There are many clinical trials conducted to try to find the best pharmacotherapy to achieve this goal. The aim was to compare the main international randomized clinical trials on hypertension in people older than 50 years. Methods: Literature qualitative review of randomized clinical trials selected from PubMed and UpToDate in people older than 50 years, from 1985 until 2020. The clinical trials conducted during this period show variability in the drugs used, the inclusion criteria for blood pressure figures, and the consideration or not of other vascular risk factors (smoking, obesity, lipid disorders, diabetes, and physical inactivity). Results: Of the 8334 articles found, 19 of them fulfilled the inclusion and exclusion criteria that involved 202,638 people. The main findings of each investigation were grouped as follows: incidence of non-cardiovascular death, death of cardiovascular origin, coronary heart disease, cerebrovascular disease, renal failure, and hypertensive retinopathy. In all patients, blood pressure figures were reduced, although this did not always lead to statistically significant differences in morbidity and/or mortality risk reduction. Twelve of them found risk reduction as an effect of reduced blood pressure. Conclusions: Randomized clinical trials conducted on hypertension in people older than 50 years of age show variability in the inclusion criteria. Variability in the antihypertensive drugs used was observed in this population. Blood pressure figures were reduced in all cases, although without statistically significant differences in morbidity and/or mortality risk reduction.

## 1. Introduction

Hypertension (HT) is one of the most important cardiovascular risk factors, particularly in the United States. Second only to smoking, HT was the modifiable risk factor responsible for the largest number of deaths from cardiovascular disease [1]. Moreover, in 2010, it was the main cause of death and disability-affected life years worldwide [2,3]. The prevalence of HT in adults over 18 years has been shown to range between 30 and 45%, in the case of stage 1 HT (systolic blood pressure: 140–159 mmHg, and diastolic blood pressure: 90–99 mmHg) [2].

Studies have demonstrated the correlation between middle and old age, blood pressure (BP), and death from cardiovascular disease, as well as providing evidence of the parallel increase between cause and effect, with values starting at 115 mmHg for systolic blood pressure and 75 mmHg for diastolic blood pressure [4]. When BP is reduced, mortality rates and the risk of cardiovascular complications likewise decrease [5].

Since 1948, when the British epidemiologist Sir Austin Bradford Hill (1897–1991) demonstrated the causal relationship between habitual smoking and lung cancer, more than 150,000 randomized clinical trials have been carried out in different fields of health sciences, these being necessary for licensing the use of new drugs [6]. The most significant clinical trials began to take place in the 1980s; however, a large number of these trials were carried out in the first decade of this century. In a significant number of these clinical trials, the goal was to determine the reduction in the risk of various symptoms of cardiovascular disease by reducing blood pressure, using different types of antihypertensive drugs, or a mixture of them where necessary.

At this time, there is no review in the scientific literature of the main clinical trials on HT that analyzes them from a qualitative perspective. The aim of this review is to compare the main international randomized clinical trials on hypertension in adults over 50 years of age, taking into account the pharmaceutical treatment used.

## 2. Experimental Section

A literature qualitative review of randomized clinical trials was carried out. The search strategy followed was a search in PubMed (National Library of Medicine, Bethesda, MD, USA) of clinical trials published from January 1985 to January 2020. Search terms were distributed into three blocks: (a) health problem: “hypertension”, (b) study design: “randomized controlled trial”, (c) types of treatment: “antihypertensive agents”, “angiotensine II type 2 receptor blockers”, “angiotensine II type 1 receptor blockers”, “angiotensin-converting enzyme inhibitors”, “calcium channel blockers”, “adrenergic beta-antagonists”, “diuretics”. Furthermore, the search was completed in UpToDate (Wolters Kluwer Health, Holland).

**Figure 1:**
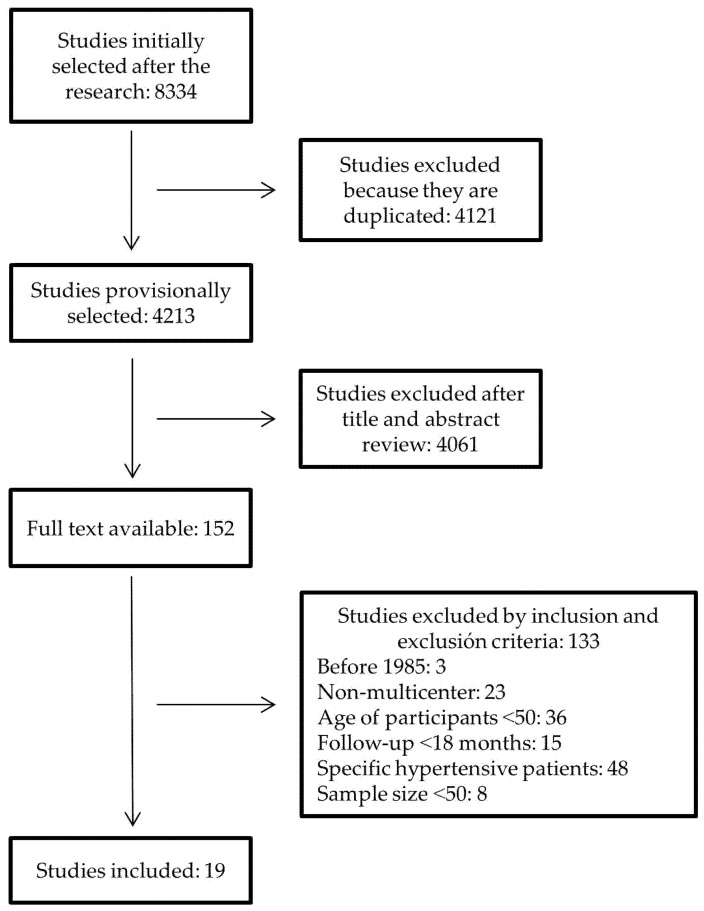
Flowchart of selection of randomized clinical trials.

After removing duplicate studies in the initial search, a preliminary selection was made by reading the title and summary of each study. The original studies of those selected were obtained and subjected to an in-depth reading in order to choose those that fulfilled inclusion and exclusion criteria. Of the total number of clinical trials identified, those selected had been published after 1985, had a sample size of at least 50 persons, and the interventions that were performed were multicenter and involved one, two, or more drugs to achieve normal BP figures. Those studies in which participants were younger than 50, that had a follow-up of less than 18 months, or that focused on particular hypertensive patients who also had other diseases such as diabetes or chronic kidney disease were ruled out. All the information gleaned from the documents was recorded in data collection sheets designed for this purpose. Observational studies, revised articles, and follow-up assessments of subgroups of primary studies were excluded. Figure 1 shows the flow chart of the selection process.

The “vote counting” method was used to display the results obtained due to the great variability of treatments used, of BP figures, cardiovascular risk factor criteria, and results of clinical trials performed with samples of individuals whose age was ≥50 years.

The investigation conforms to the principles outlined in the Declaration of Helsinki [7].

## 3. Results

The search initially identified 8334 clinical trials, of which 19 were eventually selected to be included in this review [8,9,10,11,12,13,14,15,16,17,18,19,20,21,22,23,24,25,26], involving 202,638 persons from all five continents. The features of the studies and the patients they involved can be seen in Table 1. 

**Figure 2:**
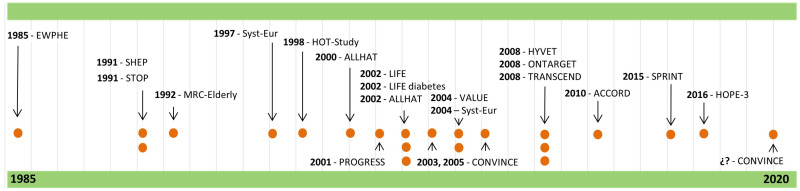
Temporal distribution of randomized clinical trials.

The number of participants in the different clinical trials ranged between 840 in the EWPHE (European Working Party on High blood pressure in Elderly) trial [8], from different European countries, and 33,357 in the ALLHAT (Antihypertensive and Lipid-Lowering treatment to prevent Heart Attack Trial) in 2002 [17], from different North American origins. The geographical and temporal distribution of the studies was: European countries (*n* = 5) [8,10,11,12,20] during the second half of the 1980s and of the 1990s; North American countries (*n* = 4) [9,14,17,24] since the beginning of the 1990s; and countries from different continents (*n* = 9) [13,15,16,18,19,21,22,23,25] from the late 1990s to the present (Figure 2. In this figure, the dots symbolize the number of clinical trials published each year, and the arrows link the dots to the names of the clinical trials.).

Raised isolated systolic BP was the criterion for participating in the SHEP (Systolic Hypertension in the Elderly Program) [9], Syst-Eur (Systolic hypertension in Europe) [12,20], LIFE (Losartan Intervention For Endpoint reduction in hypertension study) [16], and HYVET (Hypertension In the Very Elderly Trial) [21] studies (*n* = 5) as well as in the SPRINT (Systolic blood PRessure INtervention Trial) [24] trial and HOPE-3 (Heart Outcomes Prevention Evaluation) [25], albeit with some additional cardiovascular risk factors (*n* = 2). The criterion for participating in HOT (Hypertension Optimal Treatment study) [13] (*n* = 1) was having high diastolic BP, while for the studies EWPHE [8], MRC-Elderly (Medical Research Council in Elderly) [11], STOP (Swedish Trial in Old Patients with hypertension) [10], and VALUE (Valsartan Antihypertensive Long-term Use Evaluation) [19], the criterion was the elevation of both the systolic and diastolic BP values (*n* = 4). This last criterion was required, along with having other cardiovascular risk factors (CVRFs), in the studies ALLHAT [14,17] and CONVINCE (COlchicine for preveNtion of Vascular INflammation in non-CardioEmbolic stroke) [18] (*n* = 3). The criterion for participating in PROGRESS (Perindopril pROtection aGainst REcurrent Stroke Study) [15], ONTARGET (ONgoing Telmisartan Alone and in combination with Ramipril Global Endpoint Trial) [22], TRANSCEND (Telmisartan RANdomised assessment Study in aCE iNtolerant subjects with cardiovascular Disease) [23], and new CONVINCE [26] studies was having suffered some type of cardiovascular disease (*n* = 4).

The main results that were taken into account were: death due to cardiovascular causes or any other causes [8,10,11,12,13,16,17,18,19,20,22,23,24,25,26] (*n* = 15), occurrence of fatal or non-fatal cardiovascular disease from cardiac causes [8,10,11,12,13,14,16,17,18,19,20,22,23,24,25,26] (*n* = 18), and occurrence of fatal or non-fatal cardiovascular disease of cerebral origin [8,9,10,11,12,13,15,16,18,20,21,22,23,24,25,26] (*n* = 16) due to renal failure [12,20] (*n* = 2) or due to hypertensive retinopathy or retinal haemorrhage [8,12,20] (*n* = 3). Some of them focused exclusively on fatal or non-fatal strokes, such as SHEP [9], PROGRESS [15], and HYVET [21] (*n* = 3), while ALLHAT [14] (*n* = 1) centered its principal results exclusively on the occurrence of fatal or non-fatal coronary disease.

According to the conclusions of the trials, the studies ALLHAT [17], CONVINCE [18], VALUE [19], ONTARGET [22], TRANSCEND [23], and HOPE-3 [25] (*n* = 6) did not show statistically significant differences in the results between the intervention group and the control group. STOP [10], HYVET [21], and SPRINT [24] (*n* = 3) obtained a 21–43% [10,21] overall reduction in risk of mortality. EWPHE [8], LIFE [16], and SPRINT [24] studies (*n* = 3) showed a 13–38% [8,16] reduction in cardiovascular mortality risk. EWPHE [8], SHEP [9], MRC-Elderly [11], Syst-Eur [12,20], HOT [13], ALLHAT [14], PROGRESS [15], LIFE [16], and SPRINT [24] (*n* = 10) showed a 13–60% [8,16] reduction in cardiovascular morbidity risk. Finally, SHEP [9], STOP [10], MRC-Elderly [11], Syst-Eur [12,20], PROGRESS [15], LIFE [16], and SPRINT [24] (*n* = 8) showed a 13–47% [10,16] reduction in the risk of stroke.

Unlike ALLHAT [17], CONVINCE [18], VALUE [19], ONTARGET [22], TRANSCEND [23], and HOPE-3 [25] (*n* = 6), which did not demonstrate any statistically significant differences between intervention and control groups, the PROGRESS [15] and SPRINT [24] studies (*n* = 2)—which managed to reduce systolic BP to a maximum value of 135 mmHg after five years of follow-up—were able to reduce stroke risk by 28% and 25%, respectively, and cardiovascular morbidity by 26% and 25%, respectively. The studies ALLHAT [14] and HYVET [21] (*n* = 2), which managed to reduce systolic BP values to 136–140 mmHg, demonstrated a 25% reduction in cardiovascular morbidity and a 21% reduction in overall mortality risk, respectively. For their part, those studies in which systolic BP figures were only reduced to 141–145 mmHg [8,9,13,16,20] (*n* = 5) were able to reduce cardiovascular morbidity risk by 13% (Syst-Eur [20]) and 60% (EWPHE [8]), as well as stroke risk by 13% (LIFE [16]) and 36% (SHEP [9]). Finally, the studies that reduced systolic BP to values higher than 145 mmHg [10,11,12] (*n* = 2) achieved a 25–43% [10,11] reduction in stroke risk.

Regarding the relationship shown between risk reduction and diastolic BP values at five years of follow-up, only SHEP [9] and SPRINT [24] (*n* = 2) managed to reduce those values to 66–70 mmHg, resulting in a 25–32% [9,24] reduction in cardiovascular morbidity risk and a 25–36% [9,24] reduction in stroke risk. A greater number of studies [11,12,14,15,20,21] (*n* = 6) reduced diastolic BP figures to 76–80 mmHg, and as a result showed reductions in cardiovascular morbidity risk of between 15% (Syst-Eur [20]) and 31% (Syst-Eur [12]), as well as a reduction in stroke risk that varied between 25% (MRC-Elderly [11]) and 42% (Syst-Eur [12]). However, the studies (*n* = 4) that did not manage to lower diastolic BP figures further than 81 mmHg [8,10,13,16] showed more uneven results, with a reduction in overall mortality risk of between 13% (LIFE [16]) and 38% (EWPHE [8]) and in morbidity from cardiovascular causes of between 13% (LIFE [16]) and 60% (EWPHE [8]).

Regarding the average age of the participants that took part in each of these studies, the clinical trials [13,15] in which the average age was under 65 revealed a reduction in cardiovascular morbidity risk of between 15% (HOT [13]) and 26% (PROGRESS [15]) (*n* = 2). For its part, when the average age of the participants [14,16,24] (*n* = 3) was between 66 and 69, there were reductions in cardiovascular morbidity risk that ranged from 13% (LIFE [16]) to 25% (ALLHAT [14] and SPRINT [24]). When the average age rose to 70–74 years [8,9,11,12,20] (*n* = 5), the results indicated reductions in cardiovascular morbidity of between 15% (Syst-Eur [20]) and 60% (EWPHE [8]), as well as reductions in stroke risk of between 25% (MRC-Elderly [11]) and 42% (Syst-Eur [12]). Finally, if the average age was over 75, there was consequently a reduction in overall mortality of between 21% (HYVET [21]) and 43% (STOP [10]) (*n* = 2).

## 4. Discussion

The first clinical trials—which were carried out in the 1980s and early 1990s and involved people who had high BP levels, both systolic and diastolic—demonstrated a reduction in the risk of overall mortality, cardiovascular morbidity, and fatal or non-fatal stroke [8,10]. In 1991, SHEP [9] showed, on a large scale, that the treatment of isolated systolic HT reduced the risk of stroke by 36% (*p* < 0.01) and of cardiovascular events in general by 32% (*p* < 0.05) when a double antihypertensive treatment of chlorthalidone and atenolol was administered. Subsequently, several studies were carried out, SPRINT [24] and HOPE-3 [25] being the most recent, but they only dealt with participants who also had other CVRFs. However, prior to this, the study carried out by the Veterans Administration study group showed that the use of a triple antihypertensive therapy also reduced cardiovascular complications by 46% [27]. In addition, the latter was designed as a multicenter study involving health professionals and hypertensive patients from countries belonging to all five continents. For its part, the VALUE [19] study was also carried out with the participation of professionals and individuals from all five continents, but in this case patients with both systolic and diastolic hypertension, were included. In this sense, the HOT [13] study was the only one that demonstrated a 15% reduction in the risk of cardiovascular events (*p* = 0.03) and a 36% reduction in acute myocardial infarction (AMI) (*p* = 0.002) in persons from Europe, Asia, and America; participants were treated with felodipine and salicylate, plus an ACE inhibitor and a beta-blocker if necessary, in persons with isolated diastolic hypertension. Nevertheless, this treatment did not have any effect on the risk of stroke. More recently, the latest data from the SPRINT study [28] indicate that a marked decrease in blood pressure in hypertensive persons who do not have a history of diabetes mellitus, stroke, or congestive heart failure may increase cardiovascular risk if the diastolic BP falls by ≤55 mmHg.

All the clinical trials analyzed compare a drug treatment with a placebo or other drugs. In every case, a decrease in BP can be seen, leading to a reduction in cardiovascular mortality and morbidity risks. This is true for diuretic drugs as well as ACE inhibitors, ARBs, beta-blockers, and calcium antagonists, whether taken individually or mixed together in different combinations. In this regard, the 2016 European Guidelines on Cardiovascular Disease Prevention in Clinical Practice [29] does not classify these drugs according to age or sex due to lack of evidence, except for certain drugs and depending on specific conditions. Their recommendations are directed at the beginning of antihypertensive treatment with a combination of two drugs for persons with very high baseline BP or at high cardiovascular risk. On the other hand, the 2017 Guideline for the Prevention, Detection, Evaluation, and Management of High Blood Pressure in Adults [30] by the American College of Cardiology and the American Heart Association, among other associations, recommends antihypertensive treatment with two drugs from different groups in individuals with systolic BP levels of ≥140 mmHg or diastolic BP levels of ≥90 mmHg, and treatment with one drug if their systolic BP is 130–139 mmHg or their diastolic BP is 80–89 mmHg.

The criteria for inclusion in PROGRESS [15], ONTARGET [22], and TRANSCEND [23] studies (together with the new edition of CONVINCE [26]), was having previously suffered one of the following: stroke, transient ischaemic attack [15,26], coronary heart disease, peripheral artery disease, cerebrovascular attack, or diabetes mellitus [22,23]. In the first of the three studies, perindopril and indapamide were administered together, while in the second two, participants were given telmisartan and ramipril together or telmisartan on its own, respectively. Neither of these two studies showed statistically significant differences in risk reductions; however, the first did demonstrate a 28% reduction in stroke risk (*p* < 0.001) and a 26% reduction in other cardiovascular events (*p* < 0.01).

Participants over 60 years [8,9,10,11,12] of age were included in the studies conducted during the 1980s and 1990s. The HOT [13] study was the first to include individuals with HT who were aged 50 years or older. All the studies published afterwards included men and women aged 50–55, and even the PROGRESS study [15] did not take age into account as an inclusion criterion. The ongoing CONVINCE study [26] reduced the lower limit for participant age to 40 years, when these participants have suffered ischaemic cerebrovascular disease without significant disability or are at high risk of transient ischaemic attack. The SHEP [9] and Syst-Eur [12] studies of the 1990s were the first to show the benefits of reducing the risk of stroke and general cardiovascular events in persons aged 60 and over with isolated systolic HT, using an antihypertensive therapy consisting of a combination of diuretic drugs and beta-blockers in the first case, and a combination of diuretics, ACE inhibitors, and calcium antagonists in the second.

At the other end of the age spectrum, it was not until 2008 that the HYVET study [21] demonstrated the benefits of treating isolated systolic hypertension in people over 80 in European countries, China, Australia, and Tunisia by reducing the risk of death from any cause using indapamide and perindropil. Both this study and the previously mentioned SHEP study [9] in North America and the Syst-Eur study [12] in Europe showed the benefits of an antihypertensive treatment versus placebo; this contrasted with the nihilistic attitude that had hitherto existed, according to which the disease had to be left to take its own course [31,32]. Studies on the subject argued that the association of HT with cardiovascular disease risk decreases as age increases [33], and that BPs under 140/70 mmHg were associated with increased mortality in persons over 80 years old [34]. Thus, the HYVET study helped to change this trend, leading to treatment being offered to a larger segment of the population, such as the over 80s [35].

This review presents a historical recap of the main clinical trials conducted in individuals with hypertension but without other specific pathologies from 1985 to the present, taking into account the variability of pharmacological treatments used. It shows that, although most subjects achieve the target blood pressure figures, a combined antihypertensive therapy is often necessary.

## 5. Conclusions

Randomized clinical trials conducted on hypertension in people over 50 shows variability in terms of the inclusion criteria for patients and in the principal results, as well as a great variability in the antihypertensive drugs used.

In all cases, blood pressure figures could be lowered, translating into a reduction in risks, although the differences in morbidity and/or mortality reduction risks were not always statistically significant.

## Figures and Tables

**Table 1:** Characteristics of the clinical trials included.

Clinical Trial (Publication Year)	Number of Participants, Their Age, and Country of Origin	Purposes	CRITERIA for Hypertension and Other Cardiovascular Risk Factors	Antihypertensive Treatment Used	Use of Placebo	Main Results	Conclusions
EWPHE [8], (1985)	840, ≥60 years. European countries	Measure the effects of antihypertensive treatment in patients older than 60 years.	Systolic: 160–239 mmHg, Diastolic: 90–119 mmHg	Hydrochlorothiazide +Triamterene (+Methyldopa, if necessary) vs. placebo	Yes	Death, non-fatal subarachnoid haemorrhage, hypertensive retinopathy grade III or IV, dissecting aneurysm, congestive heart failure not controllable without diuretics or antihypertensive treatment, hypertensive encephalopathy, severe left ventricular hypertrophy, and a rise in blood pressure exceeding the defined limits.	38% reduction in total cardiovascular mortality (*p* = 0.023) and 60% reduction in cardiovascular morbidity.
SHEP [9], (1991)	4736, ≥60 years. United States	To establish whether an antihypertensive treatment reduces risk of fatal and non-fatal stroke in patients with isolated systolic hypertension.	Systolic: >160 mmHg, Diastolic: <90 mmHg	Chlorthalidone + Atenolol vs. placebo	Yes	Fatal stroke or not	36% reduction in stroke risk (*p* < 0.01) and 32% reduction in all cardiovascular events (*p* < 0.05).
STOP [10], (1991)	1627, 70–84 years. Sweden	To assess the ability of antihypertensive treatment to reduce the risk of non-fatal and fatal stroke, non-fatal and fatal acute myocardial infarction, and other deaths caused by cardiovascular events.	Systolic: 180–230 mmHg, Diastolic: 105–120 mmHg or ≥90 mmHg	Atenolol (+Hydrochlorothiazide and Amiloride, if necessary +Metropolol or Pindolol, if necessary) vs. placebo	Yes	Stroke, AMI, deaths from cardiovascular causes (sudden death, heart failure, and other fatal cardiovascular events)	47% reduction in stroke risk (*p* < 0.01) and 43% reduction in mortality in general (*p* < 0.01).
MRC-Elderly [11], (1992)	4961, 65–74 years. United Kingdom	To establish whether treatment with diuretic or beta blocker in hypertensive older adults reduces risk of stroke, coronary heart disease, and death.	Systolic: <200 mmHg, Diastolic: 90–109 mmHg	-Amiloride + Hydrochlorothiazide vs. placebo-Atenolol vs. placebo	Yes	Fatal and non-fatal stroke, sudden coronary death, fatal and non-fatal AMI, and death due to hypertension, to rupture or dissection of an aorticaneurysm, or to any other cardiovascular cause.	25% reduction in stroke risk (*p* = 0.04) and 17% reduction in all cardiovascular events (*p* = 0.03).
Syst-Eur [12], (1997)	4695, ≥60 years. 23 European countries	To assess whether the antihypertensive treatment reduces the rate of cardiovascular complications in isolated systolic hypertension.	Systolic: 160–219 mmHg, Diastolic: <95 mmHg	Nitrendipine and/or Enalapril and/or Hydrochlorothiazide vs. placebo	Yes	Death, stroke, retinal haemorrhage or exudates, AMI, congestive heart failure, dissecting aortic aneurysm, and renal insufficiency.	42% reduction stroke risk (*p* = 0.03) and 31% reduction in all cardiovascular events (*p* < 0.001).
HOT-Study [13], (1998)	18,790, 50–80 years. 26 countries of Europe, America and Asia	To establish the optimum target diastolic blood pressure and the potential benefit of a low dose of acetylsalicylic in the treatment of hypertension.	Diastolic: 100–115 mmHg	Felodipine (+ACE inhibitor +Beta-Blockers, if necessary) + Acetylsalicylic Acid vs. placebo	Yes	Fatal or non-fatal AMI, fatal or non-fatal stroke, and other deaths due to cardiovascular causes.	15% reduction in risk of cardiovascular events (*p* = 0.03) and 36% reduction in AMI (*p* = 0.002). No effect on stroke.
ALLHAT [14], (2000)	24,335, ≥55 years. United States and Canada	To compare the effect of doxazosin, a beta-blocker, with chlorthalidone, a diuretic, on incidence of cardiovascular diseases in patients with hypertension as part of a study of four types of antihypertensive drugs: chlorthalidone, doxazosin, amlodipine, and lisinopril.	Systolic: ≥140 mmHg, or Diastolic: ≥90 mmHg and one or more cardiovascular risk factors	Chlorthalidone vs. Doxazosin	No	Fatal coronary disease or non-fatal AMI	25% reduction in risk of other cardiovascular diseases (*p* < 0.001) in chlorthalidone group.
PROGESS [15], (2001)	6105, with no age limits Australia, Belgium, China, France, Italy, Ireland, Japan, New Zealand, Sweden, and the United Kingdom	To determine the effects of a flexible antihypertensive treatment based on perindopril and indapamide on stroke and other cardiovascular events in patients with a history of stroke or transient ischaemic attack.	Stroke or transient ischaemic attack.	Perindopril +Indapamide vs. placebo	Yes	Fatal stroke or not	28% reduction in stroke risk (*p* < 0.001) and 26% reduction in all cardiovascular events.
LIFE [16], (2002)	9193, 55–80 years. Scandinavia, the United Kingdom, and the United States	To establish whether an angiotensin II receptor blocker improves left ventricular hypertrophy beyond reducing blood pressure and, consequently, reduces cardiovascular morbidity and death.	Systolic: >160 mmHg, Diastolic: <90 mmHg and left ventricular hypertrophy	Losartan + Hydrochlorothiazide + another antihypertensive (if necessary) vs. Atenolol + Hydrochlorothiazide + another antihypertensive (if necessary)	No	Death due to cardiovascular causes, AMI, stroke	13% reduction in risk of cardiovascular death, stroke and AMI (*p* = 0.021) in losartan group.
ALLHAT [17], (2002)	33357, ≥55 years. The United States, Puerto Rico, Virgin Islands, and Canada	To determine whether treatment with a calcium channel blocker or an ACE inhibitor lowers the incidence of coronary heart disease or other cardiovascular disease events vs. treatment with a diuretic.	Systolic: ≥140 mmHg, or Diastolic: ≥90 mmHg and one or more cardiovascular risk factors	-Lisinopril vs. Chlorthalidone -Amlodipine vs. Chlorthalidone	No	Death due to coronary disease, non-fatal AMI.	No statistically significant differences in both groups.
CONVINCE [18], (2003)	16,602, ≥55 years. The United States, Canada, Europe, Mexico, Brazil, and Israel	To establish the equivalence between an extended treatment with controlled onset verapamil and a standard treatment for preventing cardiovascular disease events.	Systolic: 140–190 mmHg, Diastolic: 90–110 mmHg and one or more cardiovascular risk factors	-Verapamil vs. Atenolol -Verapamil vs. Hydrochlorothiazide	Yes	AMI, stroke, sudden death due to coronary cause or death due to cardiovascular disease.	No statistically significant differences in both groups
VALUE [19], (2004)	15,245, ≥50 years. 31 countries of America, Australia, Asia, Europe, and South Africa	To assess the efficacy of valsartan in the reduction of cardiovascular morbidity and mortality vs. amlodipine in hypertensive patients at high cardiovascular risk.	Systolic: 160–210 mmHg, and Diastolic: <115 mmHg	Valsartan + Hydrochlorothiazide (+another antihypertensive, if necessary) vs. Amlodipine + Hydrochlorothiazide (+another antihypertensive, if necessary)	No	Sudden cardiac death, fatal or non-fatal AMI, death after percutaneous coronary intervention or artery bypass, death due to heart failure, and death due to heart failure requiring hospital management.	No statistically significant differences
Syst-Eur [20], (2004)	4695, ≥60 years. 23 European countries	To assess the outcome of immediate versus delayed antihypertensive treatment in older patients with isolated systolic hypertension.	Systolic: 160–219 mmHg, Diastolic: <95 mmHg	Nitrendipine and/or Enalapril and/or Hydrochlorothiazide vs. placebo	Yes	Death, stroke, retinal haemorrhage or exudates, AMI, congestive heart failure, dissecting aortic aneurysm, and renal insufficiency.	28% reduction in stroke risk (*p* = 0.01) and 15% reduction in the rest of cardiovascular events (*p* = 0.03).
HYVET [21], (2008)	3845, ≥80 years. Europe, China, Australia, New Zealand, and Tunisia	To establish whether the antihypertensive treatment is beneficial in different fatal and non-fatal cardiovascular events in patients who are 80 years of age or older.	Systolic: ≥160 mmHg	Indapamide (+Perindopril, if necessary) vs. placebo	Yes	Fatal or non-fatal stroke	21% reduction in risk of death due to any cause (*p* = 0.02)
ONTARGET [22], (2008)	25,620, ≥55 years. 40 countries of America, Australia, Asia, Europe, and South Africa	To evaluate whether treatment based on telmisartan is superior to treatment with ramipril and whether a combination of the two drugs was superior to ramipril alone as a treatment to prevent vascular events in high-risk patients who had cardiovascular disease or diabetes mellitus but did not have heart failure.	Coronary disease, peripheral artery or cerebrovascular disease, or diabetes mellitus.	Telmisartan vs. Ramipril vs. Telmisartan+ Ramipril	No	Death from cardiovascular cause, AMI, stroke, and hospitalization for heart failure.	No statistically significant differences
TRANSCEND * [23], (2008)	5926, ≥55 years. 40 countries of America, Australia, Asia, Europe, and South Africa	To study whether extended telmisartan treatment reduces the rate of cardiovascular disease, AMI, stroke, or hospitalization for heart failure in patients with cardiovascular disease or at high risk of diabetes but did not have heart failure who are intolerant to ramipril, compared with placebo, in addition to other common therapies.	Coronary, peripheral artery or cerebrovascular disease, or diabetes mellitus.	Telmisartan vs. placebo	Yes	Death from cardiovascular cause, AMI, stroke, and hospitalization for heart failure.	No statistically significant differences
SPRINT [24], (2015)	9361, ≥50 years. The United States	To compare the benefit of treatment of systolic blood pressure to a target of less than 140 mmHg with treatment to a target of less than 120 mmHg.	Systolic: 130–180 mmHg and other cardiovascular risk factors	Intensive antihypertensive treatment based on diuretics and/or ACE inhibitors or ARBs (not both) and/or calcium antagonist vs. common standard treatment (+diuretic, if necessary).	No	AMI, acute coronary syndrome, stroke, decompensated heart failure, death from cardiovascular causes or any cause.	25% reduction in risk of AMI, acute coronary syndrome, stroke, heart failure, and death from cardiovascular causes (*p* < 0.001) and 27% reduction in death from any cause (*p* = 0.03) in intensive treatment group.
HOPE-3 [25], (2016)	12,705, men aged ≥55 years and women aged ≥65 years. America, Asia, Oceania, Europe, and South Africa	To establish whether antihypertensive treatment reduces the risk of cardiovascular disease in patients with a systolic blood pressure of <160 mmHg and an intermediate risk (≈1%) of significant cardiovascular disease.	No cardiovascular disease known but at least one cardiovascular risk factor.	Candesartan + Hydrochlorothiazide vs. placebo	Yes	Death from cardiovascular cause, non-fatal AMI, non-fatal stroke, cardiopulmonary resuscitation, heart failure, and coronary revascularization.	No statistically significant differences
CONVINCE [26]	2623 (non definitive) >40 years. European countries	To compare the efficacy of low dose colchicine plus common treatment with common treatment alone to prevent non-fatal recurrent ischaemic stroke and coronary events and death from vascular cause after transient ischaemic attack or cerebrovacular disease.	Cerebrovascular ischaemic disease without major disability or at high risk of transient ischaemic attack.	Colchicine vs. other treatment (antiplatelets, lipid-lowering agents, antihypertensives, and appropriate lifestyle).	No	Non-fatal ischaemic stroke, hospitalization for unstable angina not resulting in death, AMI, cardiac arrest, and death from cardiovascular cause.	Actually recruiting patients.

Abbreviations: ALLHAT, antihypertensive and lipid-lowering treatment to prevent heart attack trial; ARB, angiotensin II receptor blocker; CONVINCE, colchicine for prevention of vascular inflammation in non-cardioembolic stroke; EWPHE, european working party on high blood pressure in elderly; HOPE, heart outcomes prevention evaluation; HOT, hypertension optimal treatment study; HYVET, hypertension in the very elderly trial; AMI, acute myocardial infarction; ACE inhibitor, angiotensin-converting-enzyme inhibitor; LIFE, losartan intervention for endpoint reduction in hypertension study; MRC-Elderly, medical research council in elderly; ONTARGET, ongoing telmisartan alone and in combination with ramipril global endpoint trial; PROGRESS, perindopril protection against recurrent stroke study; SHEP, systolic hypertension in the elderly program; SPRINT, systolic blood pressure intervention trial; STOP, Swedish trial in old patients with hypertension; Syst-Eur, systolic hypertension in Europe; TRANSCEND, telmisartan randomised assessment study in ace intolerant subjects with cardiovascular disease; VALUE, valsartan antihypertensive long-term use evaluation. Source: Prepared by the author. * Intolerance to ramipril of ONTARGET study.
